# Implantable photonic nano-modulators open perspectives for advanced optical interfaces with deep brain areas

**DOI:** 10.1117/1.NPh.11.S1.S11512

**Published:** 2024-06-05

**Authors:** Ferruccio Pisanello, Massimo De Vittorio, Filippo Pisano

**Affiliations:** a Istituto Italiano di Tecnologia, Center for Biomolecular Nanotechnology, Arnesano, Italy; b RAISE Ecosystem, Genova, Italy; c Universtità del Salento, Dipartimento di Ingegneria dell’Innovazione, Lecce, Italy; d University of Padua, Department of Physics and Astronomy “G.Galilei”, Padua, Italy

**Keywords:** optical neural implants, optical nano-modulators, nano-optics

## Abstract

An emerging trend at the forefront of optical neural interfaces leverages the optical properties of photonic nanostructures to modulate light delivery and collection patterns in deep brain regions. This perspective article surveys the early works that have spearheaded this promising strategy, and discusses its promise towards the establishment of a class of augmented nano-neurophotonic probes.

## 1 Introduction

Optical methods for manipulating and monitoring neural activity in the mouse brain are driving a revolution in neuroscience.^1^ These strategies require delivering and collecting optical signals with a level of spatial and temporal precision that matches the scale of neuronal morphology (tens of μm for cell somas) and fast neuronal activity (ms). The technological challenges posed by these stringent requirements have been addressed in cortical regions by a flourishing set of all-optical strategies based on multi-photon stimulation and imaging systems.^2^

However, achieving a similar level of spatio-temporal precision in deep areas of the intact brain parenchyma *in vivo* is still an open challenge due to light scattering and absorption. The most common approaches to tackle this challenge consists of neural probes based on thin shanks featuring active micro-photonic components, such as μLEDs, waveguides, and photodetectors,^3^^–^^8^ and on probes based on multimode optical fibers, also featuring integrated sensors.^9^^–^^15^

To improve the control over the patterns of light delivery and collection that can be obtained in the deep brain, researchers have focused on augmenting the number of interaction sites by advancing microfabrication^16^^,^^17^ or the optical control over the fiber probes.^18^^–^^21^

**Fig. 1:**
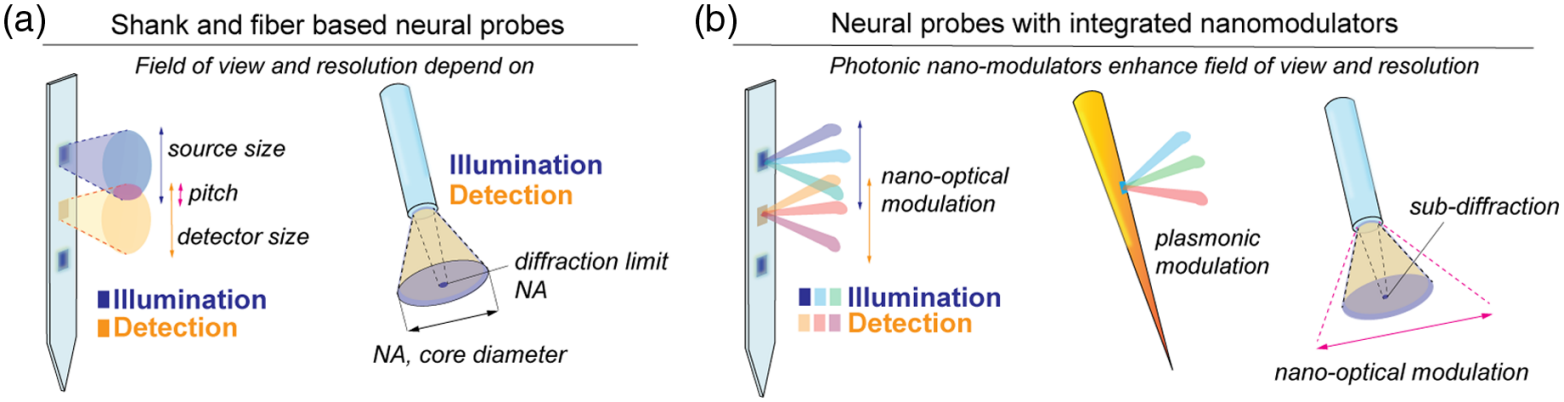
(a) The attainable patterns of illumination and detection that can be achieved with micro-photonic neural probes are governed by the geometrical dimensions of the probes, including the angular acceptance range. (b) The integration of photonic nano-modulators adds the possibility of shaping the illumination and detection patterns acting on the design of sub-wavelength elements.

Nonetheless, these strategies must deal with the limitations imposed by the intrinsic photonic properties of their components [Fig. 1(a)]. In fact, the size and pitch of integrated micro-photonic components (such as μLED or detectors) sets a limit on the achievable spatial resolution due to the Lambertian emission profile of the μLEDs combined with the effect of tissue scattering.^7^ In fiber-based neural probes, the achievable field-of-view and spatial resolution are governed by the angular acceptance of the fiber—which in turn depends on its numerical aperture (NA) and geometry.^22^^,^^23^ Fiber probes can achieve diffraction limited lateral resolution for endoscopic imaging (e.g., 0.8  μm with NA 0.37)^24^ and photostimulation,^21^ and about ∼300  μm resolution for axially resolved stimulation and photometry over volumes extending up to 2 to 3 mm.^19^^,^^25^

To surpass these limitations, an emerging trend of research is focusing on integrating nano-optical structures to add degrees of freedom in manipulating light fields at the emission and collection points. The overarching idea is to exploit optical resonances in precisely arranged array of sub-wavelength elements to control the activation and directionality of independent emission points and to shape collection patterns [Fig. 1(b)]. In the following, we will refer to these structures as photonic nano-modulators with the intent of describing a general type of microscopic optical component whose response depends on the properties of the impinging wave (wavelength, wavevector, or phase).

The paper is organized in three sections dedicated to the application of nano-modulators to longstanding challenges in the field of optical neural interfaces, such as structuring light delivery at multiple sites with a scalable approach (Sec. 2), combining multipoint light delivery with electrical recordings (Sec. 3) and achieving spatially resolved light collection with a single implant (Sec. 4).

## 2 Nano-modulators for Structuring Illumination Patterns

Controlling precise spatial and temporal patterns of illumination in deep brain regions is a requirement to fully exploit the potential of optogenetic stimulation. To this end, many approaches have proposed neural probes based on rigid or flexible shanks hosting integrated light sources, for example individually addressable μLEDs (reviewed in Ref. 26). Alternatively, micro-structured tapered optical fibers have been combined with angle-resolved light injection for restricting the illumination and collection region to a few cellular volumes at different depths.^19^^,^^27^^–^^30^ Recent works have shown that integrated light sources can be packed at high density (25  μm pitch) to achieve single-cell and ms temporal precision in optogenetic stimulation.^31^ Nonetheless, the fundamental limit on the spatial resolution remains anchored to the spatial size of the source. As a result, the scalability of LED sources is ultimately limited by the optical crosstalk between the emission volumes covered by adjacent illumination sites.

**Fig. 2:**
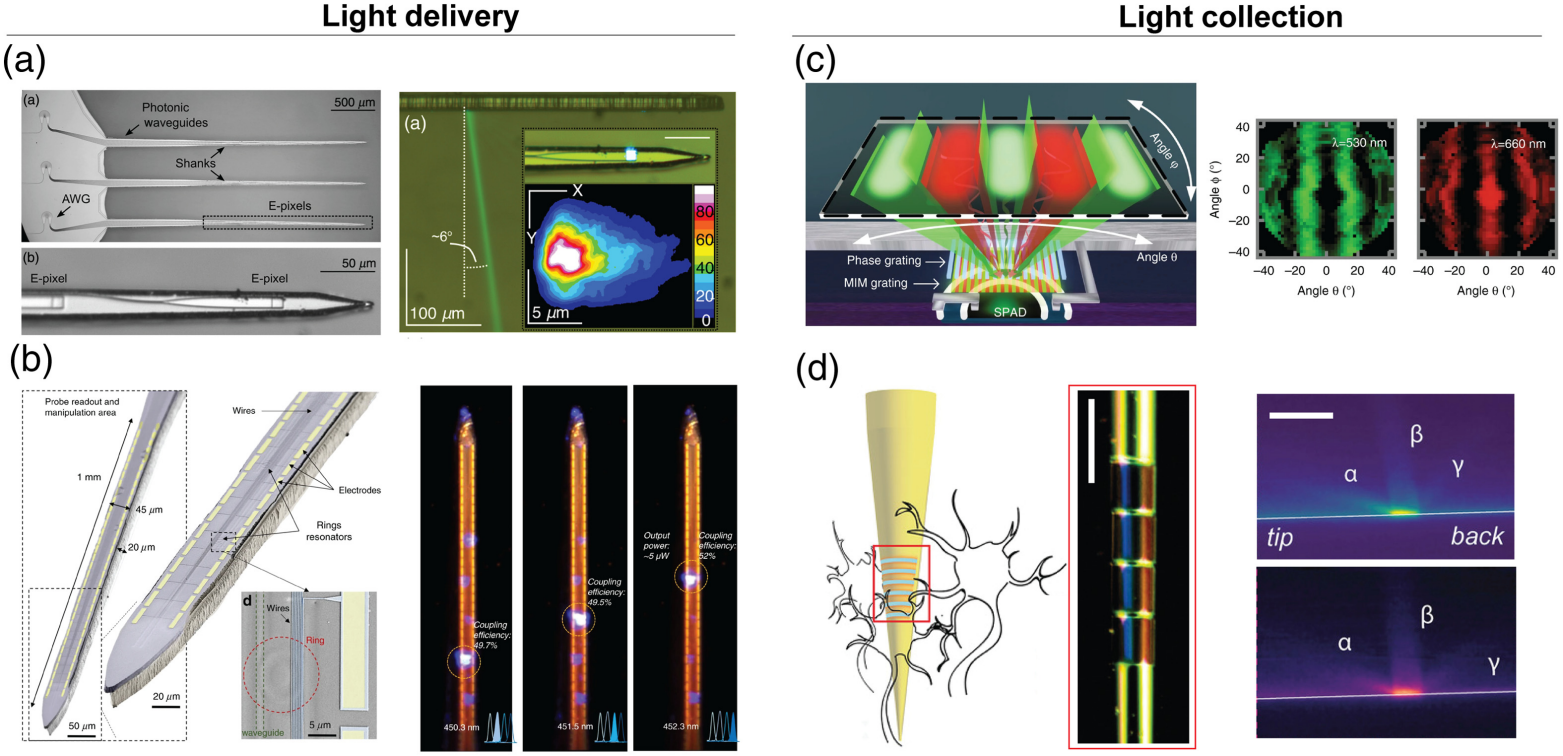
Example of neural probes using photonic nano-modulators for structuring light delivery and collection. (a) A multi-shank probe with outcoupling diffraction nano-gratings selected by wavelength modulation, adapted from Ref. 32 under CC BY 4.0. (b) An opto-electric probe using a 3D stack with ring resonators to select delivery points, adapted from Ref. 33 under CC BY 4.0. (c) Implantable lens-less imagers using angular and spectrally resolved detection through phase grating and metal-insulato-metal band pass filters adapted from Ref. 34 under CC BY 4.0. (d) Curved plasmonic nanograting select collect fluorescence light at different angles depending on the fluorophore wavelength, scale bars are 50  μm, adapted from Ref. 35 under CC BY-NC 4.0.

To circumvent this drawback, Segev et al. used a wavelength division multiplexing technique based on passive nanophotonic dielectric circuit elements to control a probe for multipoint optogenetic stimulation [Fig. 2(a)]. A prominent feature of these dielectric-based nano-modulators is that their narrow spectral resonances [∼1  nm full width at half maximum (FWHM) in Ref. 32] allow for wavelength spectral multiplexing of independent channels within the relatively large absorption spectrum of genetically encoded molecular actuators (e.g., ∼50  nm FHWM for ChR2 and GCamP6). In Seget et al.’s work, the illumination patterns were encoded in spectrally multiplexed data streams, relayed to the probe, and then de-multiplexed using arrayed waveguide gratings. Each channel was then routed to independent diffractive nano-gratings using integrated photonic waveguides, with 10 to 20  μm long grating spaced as close as 100  μm. The outcoupled beamlets had a waist of ∼30  μm at ∼200  μm from the probe.

Considering that the shanks used for dielectric outcouplers have a similar footprint to the ones hosting LED micro-emitters (mm long, 100 to 200  μm wide, and 50 to 100  μm thick), with the notable exception that nanogratings outcouplers have not been extended yet to flexible substrates, the limited space for photonic circuitry available on the shank, might hinder nano-photonic probe from reaching the same high-density now available using complementary metal-oxide-semiconductor (CMOS) architectures.^31^ To alleviate this drawback, Sacher et al. recently proposed probes that use integrated optically phased arrays to deliver optical beams that can be steered with small changes on the injection wavelength, again capitalizing on the abundance of spectral space within the absorption band of molecular actuators and reporters.^36^ At the same time, the directionality of the nano-gratings emission has been exploited to generate sheets of light at arbitrary depths in the brain, in combination with free space detection of the generated fluorescence.^37^^,^^38^

Compared with the more established μLED sources, dielectric nanogratings offer sharper and steerable beamlets with narrower spectral widths, two advantages that can potentially enable reaching a higher spatial resolution in neural stimulation using shanks.

## 3 Opto-electrical Probes with Nano-modulators

To understand the wiring of neural micro-circuitry, it is central to combine optogenetic stimulation with electrophysiological recording capabilities. Probes where light delivery points are surrounded by multiple recording micro-electrodes offer precious opportunity to monitor the response of a high number of neurons to patterned spatial stimulations at high temporal resolution (sub-ms), allowing the exploitation of spike-sorting algorithms to isolate single units. Nowadays, a number of approaches for combined optogenetic stimulation and electrophysiological recordings have been demonstrated in vivo using multifunctional optrodes,^16^^,^^39^ polymeric fibers,^9^ fiber-electrode assemblies,^40^ or micro-structured tapered fibers.^30^ Extending the electrical functionality to nanophotonic probes presented a fabrication channel that has been tackled with different approaches.

Lanzio and colleagues addressed the challenge of incorporating recording electrodes on nanophotonic circuits using staked layers housing the photonic and the electrical circuitry, exploiting wavelength modulation of passive nanophotonic elements for reconfigurable light delivery^33^ [Fig. 2(b)]. In the photonic layer, the stimulation light is distributed using a single bus waveguide connected to multiple grating out-couplers through ring resonators with narrow resonances (FWHM<1  nm). This photonic architecture allowed the integration of 64 recording electrodes and at least four excitation points on a single shank, depending on the excitation laser bandwidth.

Alternatively, Mohanty et al. put forward a device that incorporates recordings micro-electrodes alongside a rapid (μs) active circuitry of nano-photonic switches to control up to eight stimulation beams at the same wavelength.^41^ The routing network relies on cascaded 1-input-2-output Mach–Zender interference switches where the output channel is selected inducing a phase change through integrated micro-heaters connected with control electronics.

Considering the packing density of stimulation and recording sites recently achieved in high-density μLED-based optrodes (e.g., 128 stimulation points and 256 electrodes on four shanks^16^), nano-photonic optrodes have promising potential to scale up the number of excitation sites to offer an alternative for closed-loop neural stimulation and recording.

## 4 Structuring Light Collection

The approaches described above show that diffractive nano-grating outcouplers emit narrow beamlets at an angle with respect to the probe surface. Intriguingly, this property can be exploited also in “reverse,” such as using diffraction grating to extract information on the spectral content and impinging angle of the collected photons. The possibility of discriminating the wave-vector components of collected photons is an interesting strategy to increase resolution in scattering tissue while limiting the challenges linked with the integration of multiple spatially resolved detection points with image-forming optical elements.

Leveraging this concept, Taal et al. demonstrated a lens-less implantable neural imager capable of fluorescence lifetime spectroscopy^34^ [Fig. 2(c)]. The device achieves angular and spectral sensitivity by combining a phase diffraction grating (angular sensitivity) and a band-pass metal-insulator-metal Fabry–Peròt filter (spectral sensitivity) on top of metal-oxide-semiconductor (CMOS) imagers with time-gated single-photon avalanche photodiode (SPAD) pixel detectors distributed across four shanks (with 256 SPADs per shank).

Interestingly, also metallic nanostructures offer an opportunity to manipulate light delivery and collection taking advantage of the optical properties of plasmonic resonances.^42^ In this direction, we fabricated a curved plasmonic nanograting on a tapered optical fiber coated with a thin gold layer to capture light through extraordinary optical transmission (EOT), introducing a collection strategy based on wave-vector encoding^35^ [Fig. 2(d)]. As the EOT depends on the wavelength and angle of impinging light, the spectral content of the light collected through the plasmonic nanograting carries information on its original direction, regardless of its propagation in the multimode fiber.

## 5 Discussion and Conclusion

The properties of photonic nano-modulators—such as versatility, small footprint, negligible heat delivery, and bidirectional operability—have made them an attractive option to inform a novel generation of photonic neural interfaces aiming at dense stimulation and recording of brain activity.^43^

To reach this goal, the research community must surpass compelling challenges related to the fabrication and packaging of nano-photonic neural probes, their control, and their final application in highly scattering brain tissue.

Concerning the fabrication aspects, the scalability of nano-fabrication processes on neural probes based on silicon shanks supports the long-term perspective of mass manufacturing both in the front-end and back-end processes, an essential step to diminish the cost of a single device and favor widespread adoption. In this direction, a closer collaboration between research and industry partners would be desirable to ensure the repeatability standards that are, for the most, unattainable for a research laboratory.

Instead, the fabrication of photonics nano-modulators on fiber probes requires unconventional approaches, since the morphological features of fiber optics, such as the high aspect ratio of the fiber tip and the high curvature of the fiber surface, are unsuited to standard nano-patterning approaches.^44^ Nonetheless, a stream of seminal papers is tackling this challenge, showing that nano-modulators hosted the fiber tip can enhance the angular acceptance of single-mode fibers,^45^^,^^46^ a capability that might soon find application in neural investigations [Fig. 1(b)].

Another relevant challenge for the integration of photonic nano-modulators on fiber probes consists in governing the interaction between the nano-modulators and the complex patterns of intensity and angles impinging on them because of multi-modal propagation. To solve this issue, we have recently proposed a strategy to control plasmonic nanostructures fabricated on the distal tip of a multimode fiber using wavefront engineering at the proximal fiber side. Doing this, we observed that the interaction of a modal subset with a plasmonic array can generate reconfigurable foci with sub-diffraction features.^47^

Besides the technical challenges in nano-pattering and control, the effect of tissue scattering is an overarching concern on the ultimate optical performances of nano-optical collection of light from deep brain regions (signal-to-noise ratio, depth of imaging, and spatial resolution). Some authors have suggested that cell somas can be spatially localized even in the context of mesoscopic scattering regimes.^43^ At the same time, recent works have shown that computational de-mixing approaches allow retrieving time-dependent fluorescent signals from multiple sources recorded through turbid media, including multimode fibers.^48^^,^^49^ Therefore, the perspective of achieving nano-photonic collection of fluorescence signals at cellular resolution in deep brain areas has reliable foundations.

In conclusion, photonic nano-modulators promise to ease the challenge of unraveling elusive features in the physical (μm) space of brain circuits taking advantage of the photonic space in which they can be modulated (wavelength, wavevector, phase, and time). If this promise holds true, the next few years will see the rise of deep brain nano-optical neural interfaces.

## Data Availability

Data sharing is not applicable to this article, as no new data were created or analyzed.
